# From Disgust to Curiosity: Investigating Saudi University Students’ Willingness and Attitudes Toward Edible Insects as an Alternative Protein Source

**DOI:** 10.3390/insects16090963

**Published:** 2025-09-13

**Authors:** Hala Hazam Al-Otaibi, Samar Refat Alabdulmohsen

**Affiliations:** Department of Food and Nutrition Science, College of Agricultural and Food Science, King Faisal University, Al-Ahsa 31982, Saudi Arabia; 223002340@student.kfu.edu.sa

**Keywords:** entomophagy, edible insects, Saudi Arabia, Arab region, university students, willingness, alternative protein, disgust, environmental concern, food security

## Abstract

**Introduction:** With global protein demand rising, edible insects offer a nutrient-rich and environmentally sustainable alternative protein source that could help address both dietary and ecological challenges. This study investigates the attitudes, perceptions, and willingness of Saudi university students to consume edible insects (entomophagy) as an alternative protein source, with implications for public health, nutrition, and food security across the Arab region. **Methods:** A cross-sectional survey was conducted among 1711 students (34.4% men, 65.6% women) from multiple Saudi universities. The Eating Attitudes Questionnaire (EAQ) assessed disgust, interest, and attitudes toward using insect-based feed for animals, as well as measures of environmental concern, health risk beliefs, and social influences. **Results:** Significant gender differences were observed in willingness: men reported higher willingness to consume edible insects (21.1%) compared with women (8.5%). They also reported lower disgust scores, higher interest, greater environmental concern, and lower health risk beliefs. Regression analysis showed that positive attitudes toward using insects as animal feed, interest, environmental concern, and social influences were strong predictors of willingness, whereas disgust and health risk beliefs were negative predictors. Previous experience, familiarity, and religious beliefs were not significant predictors. **Conclusions:** These findings highlight the importance of targeted interventions that reduce psychological barriers and promote willingness to try edible insects, thereby contributing to Saudi Arabia’s efforts to strengthen food security and advance sustainable nutrition strategies within the kingdom and the broader Arab region.

## Simple Summary

As the world’s population grows, there is an urgent need to find new sources of protein that are both healthy and environmentally friendly. One possible option is eating insects, which are rich in nutrients and require fewer resources to produce than traditional livestock. However, people’s feelings of disgust and cultural habits often make them reluctant to try them. This study explored how young people in Saudi Arabia feel about eating insects as food. More than 1700 university students were surveyed to understand their attitudes, interests, and concerns. The results showed that men were generally more willing to try insects than women. Students who cared more about the environment, were curious about new foods, or saw insects as suitable for animal feed were more open to eating them. On the other hand, those who strongly believed insects were unsafe or felt disgusted by the idea were less willing. These findings suggest that if awareness campaigns and education reduce feelings of disgust and highlight the environmental benefits, more people may accept insects as food. This could help improve food security and sustainability in Saudi Arabia and the wider Arab region.

## 1. Introduction

The Arab region, like many regions worldwide, faces unprecedented challenges in meeting the nutritional needs of its rapidly growing population while confronting the realities of climate change, water scarcity, and limited arable land [1]. With protein deficiency affecting significant portions of the population across Arab countries, innovative approaches to sustainable nutrition are urgently needed [2]. Traditional livestock production systems contribute substantially to greenhouse gas emissions and require extensive natural resources, making them increasingly unsustainable, particularly in the resource-constrained context of the Arab region [3].

Worldwide, edible insects have become a viable alternative protein source that may simultaneously address several environmental and public health issues. Insects provide remarkable nutritional profiles, including essential amino acids, vitamins, minerals, and high-quality protein, while requiring minimal resources for production [4]. Compared with livestock production, edible insects are environmentally sustainable, help reduce pollution through the recycling of organic waste, and significantly lower greenhouse gas emissions [5].

Insects can convert two kg of feed into one kg of body weight, which is highly efficient compared with cows, which require roughly eight kg of feed to gain one kg of weight [6]. Insects also reduce pesticide use, lowering environmental pollution and pesticide residues in food, while requiring less water and feed to generate protein [5].

Embracing insects as a food ingredient could offer a promising solution to the challenge of feeding a growing global population, enhancing food security through a healthier and more sustainable protein source.

Furthermore, the use of insect-based proteins in animal feed has gained significant scientific and commercial interest, particularly as a sustainable alternative to traditional protein sources in poultry diets, such as soybean meals. Increasing scientific evidence has also encouraged research on consumer acceptance of insects in animal feed, including aquaculture and poultry farming [7,8].

In the Arab region, where food security remains a critical concern, insect protein could provide an affordable, sustainable solution to nutritional deficiencies. However, the acceptance of edible insects as food varies significantly across cultures and populations. Locusts and grasshoppers are common examples in the region; these have traditionally been eaten for their nutritional value, particularly as a protein source, and are frequently considered halal food [9].

Nearly 2000 species of edible insects are currently consumed worldwide as powder added to bread and biscuits or consumed directly (raw or cooked) [10,11]. In Europe and North America, edible insects are comparatively unknown, whereas they are widely consumed in Asia, Africa, and South America [12]. In many societies, entomophagy (the practice of eating insects) faces considerable psychological and cultural barriers, primarily rooted in disgust responses and unfamiliarity [13]. Understanding these attitudinal factors is crucial for developing effective public health interventions that promote sustainable nutrition practices.

Disgust toward entomophagy is strongly associated with the anticipated negative sensory experiences and psychological discomfort, largely driven by cultural perceptions of insects as unclean or pathogenic. In Western societies, 64% of consumers cite disgust as the primary reason for rejecting insects, compared to 28% in Thailand, where entomophagy is normalized [14]. Visual cues, such as the appearance of legs or antennae, often trigger contamination fears, reinforcing aversion even in contexts where insects are nutritionally and environmentally sustainable [14,15]. According to Liu et al. (2020) [16], Chinese participants in urban settings are increasingly receptive to edible insects, particularly when offered as processed goods like powders. Perceived awareness of nutritional advantages, willingness, familiarity, and safety perceptions of insect-based foods, including chemical contaminants (heavy metals, toxins, and pesticides), biological hazards (bacteria, viruses, and parasites), and allergens, are variables affecting acceptance.

University students will shape future food trends and are generally more receptive to new eating experiences. They tend to be open to making food choices based on personal, social, and environmental values. Their interest in food and nutrition is linked to body image and food quality and is shaped by a sense of mindfulness that fosters assertiveness and rights advocacy. They also demonstrate heightened concern for environmental change and global food insecurity. This group is particularly important for entomophagy research.

According to research conducted among Italian university students, 78.2% had heard of entomophagy, and 94% were aware of the traditional insect-feeding practices in numerous countries, indicating high knowledge levels among educated youth [7]. The excellent nutritional value of insects, especially their protein content, is acknowledged by Spanish university students. Two significant factors affecting insect food consumption are advertising and insect expertise [17]. Conversely, health- and quality-related aspects are the primary determinants of Polish university students’ readiness to purchase edible insects; they are generally reluctant to use insects as an alternative protein source [18]. Compared with Irish students, French students were more willing to consume insects; however, their primary barriers are food neophobia and perception of unpleasantness [19].

Research conducted in various cultural contexts has repeatedly shown that attitudes toward entomophagy vary by gender, with men demonstrating greater acceptance of eating insects than women. Women are more likely to associate insects with contamination and show stronger neophobic reactions, whereas men are more likely to express lower levels of disgust, be less influenced by insect species or processing methods (e.g., whole vs. powdered), and cite taste motivations for consumption [20,21]. Studies at the University of Parma have revealed that nearly all students tasted products containing insects and indicated a willingness to try other edible insects in the future, highlighting the potential of educational interventions in university settings [7].

Complex correlations between sustainability attitudes and entomophagy acceptance have been observed in recent studies among university students. Research conducted in a Middle Eastern country among Turkish university students revealed that, although they typically had negative attitudes toward entomophagy, their interest could increase if they adopted sustainable eating practices [22]. Furthermore, studies have shown that men and women are motivated by different factors: for women, social expectations play a major role, whereas for men, the desire for individuality and anti-consumption attitudes are more influential [23,24]. Despite extensive entomophagy research among university students in Western and Asian countries, studies examining attitudes toward edible insects across age groups in Middle Eastern populations remain limited, with virtually no research investigating the viewpoints of Arab university students, who are likely to play a significant role in regional food systems in the future.

Saudi Arabia, the largest economy in the Arab region, offers a valuable case for examining attitudes toward alternative protein sources. Vision 2030 prioritizes food security and environmental sustainability, yet the country’s limited arable land, scarce water, and growing protein demand pose ongoing challenges. Saudi Arabia is looking into environmentally sustainable solutions, including eight native insect orders that are high in protein and fit for human consumption, such as *Diptera*, *Orthoptera*, and *Coleoptera* [25]. Although locusts have historically been eaten throughout the country and are considered halal [26], their popularity has declined due to rapid economic expansion and dietary modernization, causing shifts from traditional acceptance to varying degrees of dislike. Strategies that align cultural traditions with current food security goals can be informed by understanding these changes and their potential to revive curiosity.

Saudi university students aged 18–24, numbering more than 4.3 million, represent the future policymakers and industry leaders of the kingdom. This demographic plays a pivotal role in advancing sustainable food transitions in Saudi Arabia and has unique potential to shift societal acceptance of alternative proteins like edible insects, which could help address two key challenges: environmental sustainability (estimated 40% water savings compared with livestock) and food security (currently 80% import-dependent) [27,28].

To our knowledge, no study has examined the psychological processes underlying the rejection or acceptance of insects, particularly the attitudes and actions of Saudi university students toward insect consumption. This study aims to examine the attitudes, perceptions, and willingness of Saudi university students to consume edible insects as an alternative protein source, with implications for broader public health and nutrition challenges in the Arab region.

## 2. Materials and Methods

### 2.1. Study Design and Participants

A cross-sectional survey study was conducted with Saudi university students over a 4-month period from October 2024 to January 2025. The study employed a convenience sampling method to recruit participants from multiple universities across different regions of Saudi Arabia. Participants were required to be Saudi nationals, aged 18–24, and enrolled in a university at the time of the study. Telegram, X, and WhatsApp were among the social media platforms used to recruit participants. A Google Forms link was employed to administer an electronic questionnaire, and respondents were encouraged via word-of-mouth to share the survey within networks to increase participation.

Before completing the survey, participants were provided with a brief sentence outlining the goals, instructions, and consent procedure, emphasizing the confidentiality and anonymity of their responses. The minimum sample size was determined a priori using G*Power 3.1 to compare two independent groups (men and women). Assuming a small effect size (Cohen’s *d* = 0.20), a significance level (α) of 0.05, and 80% power, the required sample size was approximately 393 participants per group. The final sample (n = 1711) exceeded this threshold, ensuring sufficient power even for subgroup analyses. The study was conducted according to the guidelines of the Declaration of Helsinki and approved by the Scientific Research Ethics Committee at King Faisal University (KFU) with protocol code KFU-REC-2024-OCT-ETHICS2710.

### 2.2. Data Collection Instruments

The questionnaire consisted of five main sections, each adapted from previously validated instruments. To ensure cultural relevance, a forward-backward translation process was carried out by bilingual experts. A pilot test with 20 Saudi university students (10 men, 10 women; age range 19–24 years) assessed clarity, cultural appropriateness, and feasibility, and minor wording or flow adjustments were made based on their feedback; no major structural changes were required. Pilot respondents reported that the questionnaire was clear and easy to complete, with an average completion time of 10–15 min, and were excluded from the final analysis.

*Sociodemographic characteristics*: included four questions about age, gender, family region of residence, and income.

*Consumption and familiarity*: Previous consumption of insect-based foods and exposure to others consuming insects were assessed through four questions. One question evaluated previous experience: “Have you eaten insect-based foods?” (yes/no) [20]. One question assessed familiarity: “Have you ever met or heard of someone consuming edible insects, such as locusts?” Additional questions included a social influence item: “I think I can taste edible insects if I see everyone eating them”, and a religious question: “It is forbidden and should not be consumed (not halal)” (yes/no) [29,30,31,32].

*Attitudinal measures*: The questionnaire incorporated subscales from the Eating Attitudes Questionnaire (EAQ), including the disgust subscale (EAQ-D) with five items (Cronbach’s α = 0.82). Higher scores reflect a negative attitude toward direct entomophagy. The interest subscale (EAQ-I) with three items (Cronbach’s α = 0.91); higher scores indicate a positive attitude toward direct entomophagy, and the feeding animals subscale (EAQ-F) with two items (Cronbach’s α = 0.89); higher scores reflect a positive attitude toward indirect entomophagy [33].

The EAQ was developed and validated to assess attitudes toward both direct (consumption of foods containing insect-based ingredients) and indirect entomophagy (consumption of foods from animals fed with insect-based feed). The EAQ has been translated, cross-validated across multiple languages, and used in diverse cultural contexts [33,34]. A seven-point Likert scale (1 = “I strongly disagree” to 7 = “I strongly agree”) was used to score the ten.

*Acceptance of edible insects*: was operationalized through a four-item willingness-to-consume scale. Participants rated their agreement (5-point Likert scale: 1 = “strongly disagree” to 5 = “strongly agree”) with the following statements: “I would try well-cooked edible insects served as pasta”; “I would consume flour fortified with insect-based proteins”; “I might eat insects in the future if they taste good”; “I am open to purchasing foods containing insect-derived additives or proteins” [14,32]. Total willingness scores ranged from 4 to 20, with thresholds based on the study’s mean (6.98) and standard deviation (3.35): ≤4 = unwilling, 5–11 = uncertain, and ≥12 = willing to consume edible insects as alternative protein sources (Cronbach’s α = 0.84).

*Environmental concern*: was assessed through two validated items measuring ecological awareness and perceptions of insect farming efficiency. Using a 5-point Likert scale (1 = “strongly disagree” to 5 = “strongly agree”), respondents evaluated: “I consciously attempt to minimize my environmental impact through food choices, including edible insects”, “Insect farming represents an efficient method for converting organic matter into protein” [20] (Cronbach’s α = 0.78).

*Health risk beliefs*: regarding potential risks of entomophagy were assessed using three negatively framed items: “Some individuals may experience allergic reactions from eating insects”; “Consuming insects could expose me to harmful pesticides or chemicals”; “Insects may carry dangerous microorganisms” [32]. Participants rated agreement on a 5-point Likert scale (1 = “strongly agree” to 5 = “strongly disagree”), with higher scores indicating greater perceived risks (Cronbach’s α = 0.81).

Weighted means were calculated for the seven-point and five-point Likert scale responses by assigning specific weights to each option, allowing nuanced interpretation of survey data, especially when differentiating levels of agreement or disagreement was necessary [35,36].

### 2.3. Statistical Analysis

Descriptive statistics were computed for all variables. Independent *t*-tests evaluated continuous variables between men and women, and chi-square tests assessed relationships between categorical variables. Linear regression analysis was conducted with willingness to consume insects as the dependent variable and attitudinal, psychological, and social measures (disgust, interest, acceptance of insects as animal feed, environmental concern, health risk beliefs, social influence, familiarity, religious beliefs, and previous experience) as independent variables. All analyses were conducted using SPSS Statistics, version 25.0, with statistical significance set at *p* < 0.05.

## 3. Results

The study recruited 1711 Saudi university students, comprising 589 men (34.4%) and 1122 women (65.6%). The mean age was 20.72 ± 1.875 years for men and 20.58 ± 2.088 years for women. Most participants (92.9%) reported monthly family incomes of ≤10,000 Saudi Riyals. Regional distribution varied significantly by gender, with a higher proportion of men in the Eastern region (46.5% vs. 16.4%) and a higher proportion of women in the Central region (43.9% vs. 26.5%) (Table 1).

Significant gender differences emerged from previous experiences with insect-based foods. Men reported significantly higher consumption rates (26% vs. 8.7%, *p* < 0.001), indicating greater practical familiarity with entomophagy. Despite this difference, the majority of both genders (78.3% of men and 82.3% of women) reported familiarity through knowing someone who had consumed insects, particularly locusts (*p* < 0.05).

Social influence susceptibility also differed by gender, with 37.2% of men expressing willingness to try insects if others were consuming them, compared with 16.8% of women (*p* < 0.001). Regarding religious considerations, the vast majority of participants (90.8% of men and 95.9% of women) acknowledged that entomophagy is religiously permissible (halal) in Islam, with women showing significantly higher acceptance of its religious permissibility (*p* < 0.001) (Table 2).

Analysis revealed striking gender disparities in acceptance patterns. Men demonstrated significantly higher willingness (21.1%) compared with women (8.5%) to consume insects as alternative protein sources (*p* < 0.001). Conversely, women showed greater unwillingness (41.2% vs. 26.3%), while uncertainty levels remained relatively similar between genders (52.6% for men vs. 50.4% for women) (Table 2).

Comprehensive attitudinal assessment revealed significant gender differences across all measured dimensions (*p* < 0.001). Men exhibited lower disgust scores (3.091 ± 1.89) compared with women (5.592 ± 1.39), indicating lower aversion to entomophagy. Men also demonstrated higher interest levels (5.213 ± 1.99 vs. 4.493 ± 1.15) and greater acceptance of using insects for feeding animals (4.239 ± 2.01 vs. 3.102 ± 1.49).

Environmental concern scores were higher among men (4.109 ± 1.19) than among women (3.118 ± 1.68), suggesting greater recognition of insects’ ecological benefits. Health risk perception patterns differed, with men showing lower perceived risks (2.151 ± 1.41) compared with women (3.121 ± 1.92) (Table 3).

Linear regression analysis revealed robust predictive models for both men and women (Table 4). The independent variables significantly predicted men’s willingness, *F* (9, 579) = 89.32, *p* < 0.001, which indicates that the nine factors studied had a significant impact on willingness to engage in entomophagy as an alternative protein source. The model explained 58.1% of the variance in men’s willingness (R^2^ = 0.581). For women, the independent variables significantly predicted willingness, *F* (9, 1112) = 165.14, *p* < 0.001, explaining 57.2% of the variance (R^2^ = 0.572).

For men, the strongest positive predictors were attitudes toward feeding animals with insects (β = 0.853, *p* < 0.001), interest (β = 0.530, *p* < 0.001), environmental concern (β = 0.412, *p* < 0.001), and social influence (β = 0.551, *p* < 0.001). Disgust emerged as the secondary negative predictor (β = −0.244, *p* < 0.001), and health risk beliefs were the primary negative predictor (β = −0.292, *p* < 0.001).

For women, similar patterns emerged with attitudes toward feeding animals with insects (β = 0.871, *p* < 0.001), interest (β = 0.526, *p* < 0.001), environmental concern (β = 0.432, *p* < 0.001), and social influence (β = 0.524, *p* < 0.001) as positive predictors. Health risk beliefs negatively predicted willingness among women (β = −0.366, *p* < 0.001), alongside disgust (β = −0.204, *p* < 0.001).

Notably, previous experience, familiarity, and religious considerations did not significantly predict willingness for either gender, suggesting that attitudinal factors outweigh experiential ones in determining acceptance.

## 4. Discussion

Given their nutritional qualities and ecological benefits, insects and insect-based products offer a potentially ideal alternative protein source. Several studies have explored the advantages and disadvantages of insect consumption, with one of the primary barriers being disgust [37,38]. Despite its collective dimension and importance for human health, disgust is considered an adaptive reaction that can be modified by knowledge and life experiences [39].

This study provides early insight into sex-specific differences in attitudes toward entomophagy among Saudi university students, revealing notable disparities in willingness, underlying attitudes, and perceived risks. Higher disgust responses among women students, alongside greater willingness among men, may reflect broader patterns in gendered food behaviors in Arab societies, where traditional norms and social expectations influence openness to novel foods. These findings are consistent with global research indicating that men tend to exhibit greater willingness to consume edible insects, whereas women report higher disgust sensitivity and greater concern about potential health risks [20,21].

Men in the present study showed significantly lower disgust scores compared with women. Twenty-six percent of men reported previous experience of entomophagy and willingness to consume insects, while 78.3% were familiar with insect consumption through observing or knowing someone who had eaten insects. Previous experience and familiarity are significant factors in forming positive attitudes toward edible insects, increasing acceptance [36]. In a study among young participants in western Kenya, unfamiliarity and little knowledge of edible insect species posed challenges to accepting insects as food [40]. In China, where entomophagy has a long history and tradition, unfamiliarity was the most important factor influencing university students’ unwillingness to consume insects, with only 5.6% reporting familiarity with insect-based foods [41]. Similarly to the present study findings, Laureati et al. [7] found that Italian male university students were significantly more willing than women to consume insects and significantly more ready to accept insects as feed.

University students are typically the target audience for trying new foods due to their curiosity and minimal perceived risk. Spanish university students may choose to eat insects as an alternative protein source because of their awareness of excellent nutritional value, particularly protein content [17]. However, in contrast to other studies showing generally positive attitudes among university students toward purchasing foods containing insect ingredients [42], perceived health risks remain the primary determinant of Polish university students’ willingness to consume edible insects as an alternative protein source [18]. This suggests that familiarity and positive experiences with insect consumption can reduce disgust and enhance acceptance.

Social influence showed a significant sex difference in willingness to try insects. Specifically, male respondents were more willing to taste insects if others were consuming them compared with women (*p* < 0.001). This finding indicates that male students may be more responsive to peer behavior cues, potentially reflecting greater openness to social modeling in novel food contexts. Previous studies suggest that men are often more receptive to unfamiliar protein sources and less influenced by food-related disgust, partially explaining this difference [7]. Peer observation or the perception that others are consuming novel foods significantly increases young adults’ willingness to try them, as social proof and descriptive norms reduce perceived risk and uncertainty [43].

Comparisons with international studies provide a broader context for understanding Saudi university students’ attitudes toward entomophagy using the EAQ tool. In the present study, the mean score for direct entomophagy (disgust) among men was lower (EAQ-D = 3.09 ± 1.89) than scores reported in Italian samples (EAQ-D = 4.96 ± 1.75), Chilean (EAQ-D = 4.18 ± 1.76), and Turkish university students (EAQ-D = 5.59 ± 1.39), which were comparable to women participants in our study (EAQ-D = 5.90 ± 1.75) [22,44,45,46]. Saudi students exhibited more curiosity about tasting insects, with a higher interest score (EAQ-I) among men and women comparable to Turkish (EAQ-I = 2.61 ± 2.06), Italian (3.49 ± 1.94), and Chilean students (3.46 ± 1.87) [22,44,45,46].

Acceptance of indirect entomophagy (feeding insects to animals; EAQ-F) was higher among all participants, with men showing significantly greater acceptance. This pattern aligns with findings from Italy (4.69 ± 1.82) and Chile (4.16 ± 1.49) but exceeds those reported in Turkey (3.50 ± 1.97) [22,44,45,46]. The higher acceptance among Saudi students may be explained by the perception that indirect entomophagy does not conflict with cultural or religious norms, as insects are not consumed directly by humans. This form of consumption may be considered permissible within the halal dietary laws, particularly when the livestock itself meets halal requirements. Only 100 participants (5.7%) considered eating insects non-halal. Furthermore, the growing national interest in sustainable livestock feed, driven by food security and environmental concerns, may contribute to greater receptivity. In contrast, the lower acceptance among Turkish students may reflect limited market exposure to insect-based feed and reduced integration of such products into public discourse, highlighting the role of socio-cultural framing on entomophagy acceptance. While cultural and religious context limits direct applicability to non-Arab or non-Muslim populations, the purpose of this study was also to situate Saudi perspectives within a global framework. This allows meaningful cross-cultural comparisons, highlighting both unique and shared drivers of edible insect acceptance.

Environmental concern scores were higher among men than women, potentially reflecting conventional roles and cultural expectations that encourage men to engage with broader sustainability issues. Men’s perceptions of environmental responsibility as part of leadership may also be influenced by cultural norms. Understanding these characteristics can inform initiatives to involve women more actively in sustainability programs. Similar patterns were observed among Lebanese young adults [47] and Polish university students, as well as environmental impact and nutritional composition associated with insect consumption [48]. In general, young adults, particularly university students, are more aware of environmental issues sustainable healthy eating and are more willing than older generations to pay for eco-friendly products [49].

Participants’ acceptance of entomophagy was significantly influenced by health risk perceptions related to the safety of insect-derived foods. Men reported lower health risk beliefs than women, who may perceive greater risks due to heightened sensitivity to food safety and health issues [49,50]. Research suggests that women often adopt more cautious attitudes toward novel foods, potentially influenced by evolutionary factors related to protecting themselves and their offspring. Additionally, women tend to be more risk-averse regarding health matters, which can lead to stronger concerns about the safety of novel food sources such as insects.

Linear regression analysis yielded robust models predicting willingness to consume insects as alternative protein sources for both men and women, explaining over 57% of the variance in each group. This highlights the strong influence of the attitudinal factors on entomophagy acceptance, consistent with previous research emphasizing attitudes as key determinants in novel food acceptance [13,20].

For both genders, positive attitudes toward using insects as animal feed were the strongest predictors of willingness. This supports the idea that acceptance of indirect entomophagy can shape openness to direct consumption, as prior studies link environmental and ethical concerns around sustainable feed sources to broader entomophagy acceptance [44,46]. Environmental concern and social influences further reinforce the importance of normative and ecological motivations in food choice behaviors [47].

Disgust and health risk beliefs emerged as significant negative predictors. Health risk beliefs had a stronger negative effect among men, while disgust was a slightly weaker predictor. This gender variation may reflect differences in health risk perception and emotional responses, echoing findings that women often express greater disgust sensitivity and risk aversion toward novel foods [43].

Previous experience, familiarity, and religious considerations did not significantly predict willingness for either gender, suggesting that cognitive and affective attitudes, rather than experiential factors, primarily drive entomophagy acceptance in this sample. This contrasts with some earlier studies where familiarity enhanced acceptance [41], indicating that in contexts where entomophagy remains novel, these attitudinal factors dominate. The non-significant impact of religious considerations aligns with recent research suggesting that religious beliefs influence indirect acceptance more than direct acceptance [22].

This study has several notable strengths. First, the large sample of 1711 students from multiple Saudi universities enhances the reliability of findings within the target population. Second, employing the EAQ alongside measures of environmental concern, health risk beliefs, and social influences provides a comprehensive, multidimensional understanding of factors influencing willingness to consume edible insects. Third, the study identifies both positive predictors (interest, environmental concern, social influence, and attitudes toward insect-fed animals) and negative predictors (disgust and perceived health risks), offering actionable insights for targeted interventions. Fourth, positive attitudes toward insect-fed animals across both sexes suggest that introducing insect-based feed into animal production may face minimal cultural resistance, offering a feasible pathway to indirectly promote entomophagy among environmentally conscious young adults. Fifth, significant gender differences were highlighted, enabling tailored strategies for promoting entomophagy. Focused on Saudi university students, the findings are culturally and regionally relevant, supporting local and Arab regional efforts toward sustainable food security. Overall, the study offers practical implications for educational campaigns, policy measures, and interventions to reduce psychological barriers and enhance acceptance of alternative protein sources. Finally, this study fills a critical gap by being the first to explore the psychological processes shaping Saudi university students’ attitudes and behaviors toward edible insects, offering insight into the broader Arab region.

The present study has several limitations. First, the convenience sampling may limit generalizability to other Saudi university students or other age groups in the population. Future studies should include more balanced gender representation and non-student populations to enhance generalizability. Second, the study relied on self-reported data, which could introduce social desirability or recall bias. Although measures were anonymous to reduce social desirability bias, self-reported responses remain subject to recall and acquiescence bias. Future studies should integrate behavioral experiments, such as tasting sessions, to complement self-reported attitudes. Third, with a cross-sectional design, causal relationships between attitudes, predictors, and willingness cannot be established. Longitudinal or intervention-based designs are recommended to better test these causal links. Fourth, the cultural and religious context of the participants may limit the applicability to non-Arab or non-Muslim populations; however, global social media influence may make these findings relevant beyond the local context. Fifth, insect-based products are not yet commercially available in Saudi Arabia; willingness was assessed through hypothetical scenarios, which may not fully reflect real-world behavior. Future research should include tasting interventions and simulated market conditions. Sixth, other critical determinants such as sensory preferences (taste, texture, appearance), economic affordability, and broader behavioral drivers were not measured. As shown with plant-based meat substitutes, poor sensory attributes significantly reduce acceptance; thus, future studies should incorporate sensory and market-oriented factors. Finally, although key psychological, social, and environmental factors were examined, other relevant variables such as sensory preferences, economic considerations, and broader behavioral drivers were not explored, potentially limiting the comprehensiveness of the findings.

## 5. Conclusions

This study provides the first evidence of Saudi university students’ attitudes toward edible insects, revealing a complex coexistence of curiosity and disgust. Willingness to consume was strongly driven by environmental concern, personal interest, social influence, and acceptance of indirect entomophagy (e.g., insect-based flour), while health risk perceptions and disgust functioned as primary barriers. Furthermore, men demonstrated significantly greater willingness and lower disgust than women.

These findings highlight the urgent need for culturally tailored interventions that leverage motivational factors such as environmental benefits and social acceptance through targeted awareness campaigns and tasting-based approaches designed to reduce psychological barriers. Implementing these strategies is essential for enhancing public acceptance and supporting national food security strategies under Saudi Vision 2030, as well as broader regional efforts toward sustainable protein alternatives.

For future research, efforts should include balanced gender samples, extend to non-student populations to improve generalizability, and employ sensory evaluations to capture real-world behavior. Subsequent steps should also focus on tracking changes in public willingness, implementing practical tasting sessions, and integrating entomophagy education into public health and nutrition programs across the Arab region.

## Figures and Tables

**Table 1:** Socio-demographic characteristics of the participants (n = 1711).

Variables	Men, 589 (34.4%)	Women, 1122 (65.6%)
Age Mean ± SD	20.72 ± 1.875	20.58 ± 2.088
Monthly family income, n (%)		
^a^ SR ≤ 10,000	541 (91.9%)	1048 (93.4%)
SR > 10,000	48 (8.1%)	74 (6.6%)
Region of residence, n (%)		
Eastern	274 (46.5%)	184 (16.4%)
Northern	25 (4.2%)	77 (6.9%)
Western	44 (7.5%)	134 (11.9%)
Southern	90 (15.3%)	234 (20.9%)
Central	156 (26.5%)	493 (43.9%)

^a^ Saudi Riyal.

**Table 2:** Participants’ experience, social influences, familiarity, and religious beliefs willingness of acceptance regarding entomophagy as alternative protein sources (n = 1711).

Variables	Men, 589 (34.4%)	Women, 1122 (65.6%)	*p*-Value
Experience: Have you eaten insect-based foods?			
No	436 (74%)	1024 (91.3%)	0.000 ***
Yes	153 (26%)	98 (8.7%)	
Social effects: I think I can taste insects if I see everyone eating them			
No	370 (62.8)	933 (83.2)	0.000 ***
Yes	219 (37.2)	189 (16.8)	
Familiarity: Have you ever met or heard of someone consuming insects, such as locusts?			
No	128 (21.7)	199 (17.7)	0.046 *
Yes	461 (78.3)	923 (82.3)	
Religion: It is forbidden and should not be consumed (not halal)			
No	535 (90.8%)	1076 (95.9%)	0.000 ***
Yes	54 (9.2%)	46 (4.1%)	
Willingness of acceptance consuming insects			
Willingness	124 (21.1%)	95 (8.5%)	0.000 ***
Uncertainty	310 (52.6%)	565 (50.4%)	
Unwillingness	155 (26.3%)	462 (41.2%)	

* *p* < 0.05; *** *p* < 0.001.

**Table 3:** Attitudinal factors influencing entomophagy as alternative protein sources (n = 1711).

Variables Mean ± SD	Men, 589 (34.4%)	Women, 1122 (65.6%)	*p*-Value
^a^ Disgust (EAQ-D)	3.091 ± 1.89	5.592 ± 1.39	0.000 ***
^b^ Interest (EAQ-I)	5.213 ± 1.99	4.493 ± 1.15	0.000 ***
^c^ Feeding animals (EAQ-F)	4.239 ± 2.01	3.102 ± 1.49	0.000 ***
Willingness	4.667 ± 1.87	3.412 ± 1.92	0.000 ***
Environment	4.109 ± 1.19	3.118 ± 1.68	0.000 ***
Health risk beliefs	2.151 ± 1.41	3.121 ± 1.92	0.000 ***

^a^ The disgust subscale (EAQ-D); ^b^ the interest subscale (EAQ-I); ^c^ feeding animals. (EAQ-F) *** *p* < 0.001.

**Table 4:** Predictors of the willingness to entomophagy as an alternative protein source (n = 1711).

Men, 589 (34.4%)			
Variables	β	t	*p*-value
Disgust (EAQ-D)	−0.244	−12.151	0.000 ***
Interest (EAQ-I)	0.530	19.491	0.000 ***
Feeding animals (EAQ-F)	0.853	24.698	0.000 ***
Environment	0.412	12.000	0.000 ***
Health risk beliefs	−0.292	−13.876	0.000 ***
Experience	0.986	0.893	0.372
Social effects	0.551	6.005	0.000 ***
Familiarity	0.089	0.924	0.356
Religion	−0.011	−0.083	0.934
Women, 1122 (65.6%)			
Variables	β	t	*p*-value
Disgust (EAQ-D)	−0.204	−14.098	0.000 ***
Interest (EAQ-I)	0.526	26.212	0.000 ***
Feeding animals (EAQ-F)	0.871	34.558	0.000 ***
Environment	0.432	17.802	0.000 ***
Health risk beliefs	−0.366	−11.348	0.000 ***
Experience	−0.019	−0.213	0.831
Social effects	0.524	7.253	0.000 ***
Familiarity	0.015	0.253	0.800
Religion	−0.065	−0.549	0.583

*** *p* < 0.001.

## Data Availability

The datasets used and analyzed during the current study are available from the corresponding authors on reasonable requests.

## Abbreviations

The following abbreviations are used in this manuscript:

EAQ	Eating Attitudes Questionnaire
EAQ-D	Disgust subscale
EAQ-I	Interest subscale
EAQ-F	Feeding Animals subscale

## References

[1] FAO, IFAD, UNICEF, WFP, WHO, UNESCWA. 2023. Near East and North Africa—Regional Overview of Food Security and Nutrition: Statistics and Trends. Cairo.

[2] ESCWA, Trade for Food Security and Nutrition Security in the Arab Region. Distr. LIMITED E/ESCWA/CL1.CCS/2022/WG.14/Report 5 December 2022 ORIGINAL: ENGLISH. https://www.unescwa.org/sites/default/files/event/materials/1.2Trade-for-Food-Security-and-Nutrition-Security-Arab-Region-Keulertz-ESCWA.pdf.

[3] Jaber L.S., Diehl K.E., Hamadeh S.K. (2016). Livestock and food security in the Arab region: Policy framework. Food Sec..

[4] Huis A.V., Itterbeeck J.V., Klunder H., Mertens E., Halloran A., Muir G., Vantomme P. (2013). Edible Insects: Future Prospects for Food and Feed Security.

[5] Imathiu S. (2020). Benefits and food safety concerns associated with consumption of edible insects. NFS J..

[6] Abril S., Pinzón M., Hernández-Carrión M., Sanchez-Camargo A.D.P. (2022). EIsin Latin America: A sustainable alternative for our food security. Front. Nutr..

[7] Laureati M., Proserpio C., Jucker C., Savoldelli S. (2016). New sustainable protein sources: Consumers’ willingness to adopt insects as feed and food. Ital. J. Food Sci..

[8] Bazoche P., Poret S. (2021). Acceptability of Insects in Animal Feed: A Survey of French Consumers. J. Consum. Behav..

[9] Riyaz M. (2023). Edible insects: Islamic perspectives on entomophagy and future foods: Islamic perspectives on edible insects. SHAHIH J. Islam. Multidiscip..

[10] Macombe C., le Feon S., Aubin J., Maillard F. (2019). Marketing and social effects of industrial scale insect value chains in Europe: Case of mealworm for feed in France. J. Insects Food Feed..

[11] Mancini S., Moruzzo R., Riccioli F., Paci G. (2019). European participants’ readiness to adopt insects as food. A review. Food Res. Int..

[12] Govorushko S. (2019). Global status of insects as food and feed source: A review. Trends Food Sci. Technol..

[13] Hartmann C., Siegrist M. (2017). Consumer perception and behaviour regarding sustainable protein consumption: A systematic review. Trends Food Sci. Technol..

[14] Hartmann C., Shi J., Giusto A., Siegrist M. (2015). The psychology of eating insects: A cross-cultural comparison between Germany and China. Food Qual. Prefer..

[15] Ruby M.B., Rozin P., Chan C. (2015). Determinants of willingness to eat insects in the USA and India. J. Insects Food Feed..

[16] Liu A.J., Li J., Gómez M.I. (2020). Factors influencing consumption of edible insects for Chinese participants. Insects.

[17] Cantalapiedra F., Juan-Garcia A., Juan C. (2023). Perception of food safety associated with entomophagy among higher-education students: Exploring insects as a novel food source. Foods.

[18] Orkusz A., Wolanska W., Harasym J., Piwowar A., Kapelko M. (2020). Consumers’ attitudes facing entomophagy: Polish case perspectives. Int. J. Environ. Res. Public Health.

[19] Ranga L., Vishnumurthy P., Dermiki M. (2024). Willingness to consume insects among students in France and Ireland. Ir. J. Agric. Food Res..

[20] Verbeke W. (2015). Profiling participants who are ready to adopt insects as a meat substitute in a Western society. Food Qual. Prefer..

[21] Megido R.C., Gierts C., Blecker C., Brostaux Y., Haubruge É., Alabi T., Francis F. (2016). Consumer acceptance of insect-based alternative meat products in Western countries. Food Qual. Prefer..

[22] Duman E., Keser A. (2025). Entomophagy Attitudes Among Turkish Generation Z University Students: A Scale Validation and Path Analysis Model for Sustainable and Healthy Dietary Choices. Food Sci. Nutr..

[23] Kasza G., Izsó T., Szakos D., Nugraha W.S., Tamimi M.H., Süth M. (2023). Insects as food-changes in participants’ acceptance of entomophagy in Hungary between 2016 and 2021. Appetite.

[24] Florença S.G., Correia P.M., Costa C.A., Guiné R.P. (2021). Edible insects: Preliminary study about perceptions, attitudes, and knowledge on a sample of Portuguese citizens. Foods.

[25] Sharawi S.E. (2024). Exploring the various orders of edible insects in the Kingdom of Saudi Arabia as a safe and sustainable food alternative: A comprehensive review. J. Appl. Nat. Sci..

[26] Abdel-Dayem M.S., El-Ghiet U.M.A., Elsheikh T.M., Elgharbawy A.A., Al-Fifi Z.I., Aldhafer H.M. (2020). The first survey of the beetles (Coleoptera) of the Farasan Archipelago of the southern Red Sea, Kingdom of Saudi Arabia. ZooKeys.

[27] USDA Foreign Agricultural Service (2024). Exporter Guide Annual, Report SA2024-0007.

[28] Elmulthum N.A., Zeineldin F.I., Al-Khateeb S.A., Al-Barrak K.M., Mohammed T.A., Sattar M.N., Mohmand A.S. (2023). Water Use Efficiency and Economic Evaluation of the Hydroponic versus Conventional Cultivation Systems for Green Fodder Production in Saudi Arabia. Sustainability.

[29] Orsi L., Voege L.L., Stranieri S. (2019). Eating edible insects as sustainable food? Exploring the determinants of consumer acceptance in Germany. Food Res. Int..

[30] Bisconsin-Júnior A., Rodrigues H., Behrens J.H., da Silva M.A.A.P., Mariutti L.R.B. (2022). Food made with edible insects: Exploring the social representation of entomophagy where it is unfamiliar. Appetite.

[31] Hopkins I., Farahnaky A., Gill H., Newman L.P., Danaher J. (2022). Australians’ experience, barriers and willingness towards consuming edible insects as an emerging protein source. Appetite.

[32] Szlachciuk J., Żakowska-Biemans S. (2024). Breaking the taboo: Understanding the relationship between perception, beliefs, willingness to eat insects, and food neophobia among Polish adults. Foods.

[33] La Barbera F., Verneau F., Videbæk P.N., Amato M., Grunert K.G. (2020). A Self-Report Measure of Attitudes toward the Eating of Insects: Construction and Validation of the Entomophagy Attitude Questionnaire. Food Qual. Prefer..

[34] Videbæk P.N., Grunert K.G. (2020). Disgusting or Delicious? Examining Attitudinal Ambivalence towards Entomophagy among Danish Consumers. Food Qual. Prefer..

[35] Zielińska E., Zieliński D., Karaś M., Jakubczyk A. (2020). Exploration of consumer acceptance of insects as food in Poland. J. Insects Food Feed..

[36] Tolve R., Zanoni M., Sportiello L., Musollini S., Tchuenbou-Magaia F.L., Favati F. (2025). From fear to fork—Exploring food neophobia and the inclination towards entomophagy in Italy. Int. J. Food Sci. Technol..

[37] Hartmann C., Siegrist M. (2018). Development and validation of the Food Disgust Scale. Food Qual. Prefer..

[38] Cicatiello C., De Rosa B., Franco S., Lacetera N. (2016). Consumer approach to insects as food: Barriers and potential for consumption in Italy. Br. Food J..

[39] Rozin P. (2002). Human food intake and choice from biological, psychological, and cultural perspectives. Food Selection: From Genes to Culture.

[40] Owidi E., Asoka G., Waga E., Ochieng A., Kawaka F. (2025). Consumer attitudes and perceptions on consumption of edible insects among communities in western Kenya. PLoS ONE.

[41] Tian H., Chen J. (2025). Association of food neophobia and food disgust with the willingness, benefits, and risks of insect food consumption among Chinese university students. Front. Nutr..

[42] Mikulec A.T., Platta A.M., Radzyminska M., Ruszkowska M., Mikulec K., Suwała G., Kowalski S., Kowalczewski P.Ł., Nowicki M. (2024). Attitudes and purchase intentions of polish university students towards food made from insects A modelling approach. PLoS ONE.

[43] Ordoñez López M.F., Ghnimi S., Liu C. (2023). Willingness to consume insect-based food in France: Determinants and consumer perspectives. LWT.

[44] La Barbera F., Verneau F., Amato M., Grunert K.G., Schnettler B. (2021). Acceptance of Insect-Based Food in Chile: Evidence From a Survey Using the Entomophagy Attitude Questionnaire (EAQ). Food Qual. Prefer..

[45] La Barbera F., Amato M., Fasanelli R., Verneau F. (2021). Perceived Risk of Insect-Based Foods: An Assessment of the Entomophagy Attitude Questionnaire Predictive Validity. Insects.

[46] Sogari G., Riccioli F., Moruzzo R., Menozzi D., Sosa D.A.T., Li J., Liu A., Mancini S. (2023). Engaging in Entomophagy: The Role of Food Neophobia and Disgust Among Insect and Noninsect Eaters. Food Qual. Prefer..

[47] Boustani N.M., Guiné R.P. (2024). Exploring innovative food in a developing country: Edible insects as a sustainable option. Open Agric..

[48] Platta A., Mikulec A., Radzyminska M., Kowalski S., Skotnicka M. (2024). Willingness to consume and purchase food with edible insects among generation Z in Poland. Foods.

[49] Dragolea L.L., Butnaru G.I., Kot S., Zamfir C.G., Nuță A.C., Nuță F.M., Cristea D.S., Ștefănică M. (2023). Determining factors in shaping the sustainable behavior of the generation Z consumer. Front. Environ. Sci..

[50] Guine R.P., Florença S.G., Costa C.A., Correia P.M., Boustani N.M., Matran I., Ferreira M. (2024). Participants’ perceptions about edible insects’ nutritional value and health effects: Study involving 14 countries. Animals.

